# Evaluation of Serum Iron and Platelet Parameters in Dogs

**DOI:** 10.3390/ani15243613

**Published:** 2025-12-15

**Authors:** Charlotte Lubbers, Tim Andre M. Corvers, Charlotte Dye

**Affiliations:** 1 Southern Counties Veterinary Specialists, Hampshire BH24 3JW, UK; 2 Clinic for Small Animal Medicine, University of Zurich, CH-8006 Zurich, Switzerland; 3 Pride Veterinary Centre, Derby DE24 8HX, UK; charlotte.dye@scarsdalevets.com

**Keywords:** iron deficiency, anaemia, thrombocytosis, gastrointestinal disease, neoplasia

## Abstract

In humans, iron deficiency is commonly associated with reactive thrombocytosis, which has clinically relevant consequences. To investigate the relationship between serum iron parameters and platelet indices in dogs and to assess for associations between iron parameters and disease pathology, the clinical records of 141 dogs with concurrent serum iron panel and complete blood count (CBC) from 2010 to 2023 were retrospectively reviewed. In total, 86 (61%) dogs had low serum iron concentrations, with 56/86 (65%) having functional and 30/86 (35%) having absolute iron deficiency. Seventy-seven (91.7%) of the dogs with iron deficiency were anaemic and 22% (19/86) had thrombocytosis. Dogs with serum iron < 10 μmol/L had higher platelet count distributions (*p* = 0.003) and were more likely to have platelet counts >400 × 10^9^/L (*p* = 0.013). Platelet counts did not differ between dogs with functional versus absolute iron deficiency. Dogs with neoplasia had lower serum iron distributions than those with inflammatory or other disease pathologies (*p* = 0.001) and dogs with serum iron <10 μmol/L were more likely to have neoplasia (*p* = 0.001, OR 5.1). In conclusion, both functional and absolute iron deficiency appear to be associated with thrombocytosis in dogs, as seen in humans. Additional studies are required to assess whether iron-deficiency-related thrombocytosis carries diagnostic, monitoring, prognostic, or therapeutic utility in dogs.

## Simple Summary

In humans, iron deficiency is commonly associated with elevation of blood platelets, which can lead to pathologic clot formation. To investigate whether similar events occur in dogs, the clinical records of 141 dogs that had undergone concurrent testing for blood iron and platelet parameters were reviewed. Iron parameters were also compared between dogs with different disease entities. The results show that dogs with low blood iron concentrations were more likely to have elevated platelet counts. The results also document that dogs diagnosed with cancer had lower blood iron concentrations than those with other conditions such as inflammatory or immune-mediated disease. In conclusion, this study shows that, as in humans, dogs seem to have higher platelet counts when they suffer from iron deficiency. Further studies are needed to investigate whether this has clinical consequences and whether iron deficiency in dogs might be beneficial for diagnosing or monitoring certain diseases.

## 1. Introduction

Iron deficiency is the most common cause of anaemia in humans [1] and is a frequent cause of anaemia in dogs and cats [2,3,4,5]. It is usually found as a consequence of chronic blood loss, predominantly as a result of gastrointestinal haemorrhage [3,5,6]. Other causes of absolute iron deficiency include intestinal malabsorption and, rarely, nutritional deficiency in animals fed home cooked diets [4,7]. Conversely, functional iron deficiency represents an inability to utilise adequate body iron stores, and is associated with inflammatory disease, renal disease, hepatic disease, and exogenous erythropoietin administration [4,7,8,9].

Reactive thrombocytosis occurs as a result of enhanced physiological production of platelets secondary to an underlying disease [10]. It is a common haematologic abnormality with a variety of aetiologies including infection, chronic inflammation, iron deficiency, acute blood loss, tissue damage, neoplasia, and drugs in both dogs [11,12] and humans [13]. Iron deficiency accounts for 18% of all human patients with thrombocytosis, and there is a significant association of platelet count with both absolute and functional iron deficiency [13,14]. While the mechanism is not well defined, iron deficiency induced thrombocytosis is independent of other risk factors [13,15,16]. In humans, most patients with iron deficiency, develop mild-to-moderate thrombocytosis (on average, twice that of control groups); however, a minority (7–13%) develop extreme thrombocytosis (>950 × 10^9^/L) [10]. Reactive thrombocytosis is associated with concurrent platelet activation [15,16,17] and, in humans, there is accumulating evidence that this has significant clinical relevance, particularly in the setting of iron deficiency [17,18,19,20]. Indeed, there are multiple reports of thromboembolic complications in patients with iron deficiency induced thrombocytosis [17,18,21].

In human medicine, thrombocytosis has diagnostic, monitoring, therapeutic, and prognostic implications for a variety of diseases [10,14,20]. In contrast, the incidence, disease associations and clinical significance of thrombocytosis in veterinary species is unclear. Several studies have suggested that, in dogs, thrombocytosis is most commonly associated with neoplasia, inflammatory disease and endocrine disorders [11,12,22,23], and an increased incidence of concurrent anaemia and thrombocytosis has been documented in ageing dogs [24], in dogs with neoplasia [11,12,22], and in dogs with chronic enteropathy [2]. However, although acknowledged in some of these studies, the incidence of iron deficiency and any correlation with platelet count has not been comprehensively established. For example, a previous study comprising 81 anaemic dogs found higher platelet counts in those with iron deficiency [5], while another comprising 22 dogs with chronic enteropathy found no difference in serum iron concentration between dogs with thrombocytosis and those without [2].

The primary aims of this study were to assess the prevalence of thrombocytosis in dogs with iron deficiency, and to evaluate for possible associations of iron and platelet parameters with specific disease entities. The hypothesis was that, as in humans, platelet counts would be increased in dogs with iron deficiency.

## 2. Materials and Methods

The clinical records of dogs with concurrent serum iron panel and complete blood count (CBC) results performed at a large veterinary referral and first-opinion hospital over a 13-year period from 2010 to 2023 were reviewed. The CBC was considered to be concurrent if it was performed within 24 h of the iron panel submission. All CBC samples were run in-house (IDEXX ProCyte Dx Haematology Analyser, Hoofddorp, The Netherlands), and all automated platelet counts were verified by in-house lab technicians using Diff-Quik stained fresh blood smears. Serum iron panels (total serum iron, transferrin saturation, total iron binding capacity) and serum cobalamin were run externally at a single reference laboratory using the Beckman Coulter method for iron parameters. Transferrin saturation (%) was calculated using the following formula: total serum iron/total iron binding capacity × 100. Serum cobalamin was measured using competitive chemiluminescence immunoassay (CLIA). These methodologies remained unchanged over the study period.

Data collection included signalment (sex, neuter status, breed, age), diagnosis, previous medications, haematocrit, platelet count, mean platelet volume (MPV), serum iron concentration, total iron binding capacity (TIBC), transferrin saturation and, where available, serum cobalamin concentration. Exclusion criteria included diagnosis of immune-mediated thrombocytopenia, primary bone marrow disease, and recent blood transfusion or chemotherapy (within 6 weeks of iron panel submission). Cavalier King Charles Spaniels were also excluded due to a high breed incidence of congenital macrothrombocytopaenia [25]. Data was collected and stored in compliance with General Data Protection Regulation (GDPR) requirements. Dogs that met the inclusion criteria were grouped according to signalment and disease categories as follows: sex (male entire [ME], male neutered [MN], female entire [FE], female neutered [FN]), age, affected body system (gastrointestinal [GI], hepatic, renal, haematologic, other), and disease pathology type (inflammatory, immune-mediated, neoplastic, other).

Statistical analysis was performed using the IBM SPSS software package, version 31.0.0.0. Sex, age, body system, and pathology type were assessed as categorical data with amalgamation of categories to optimise comparable group sizes as follows: sex (female/male), age (<6 y, 6–10 y, >10 y), and body system (GI, other). The remaining variables were expressed as both categorical and continuous data with categorisation to optimise comparable group sizes for statistical analysis as follows: serum iron (<10, 10–20, >20 μmol/L), transferrin saturation (<25, >25%), TIBC (<55, 55–70, >70 μmol/L), serum cobalamin (<300, >300 ng/L), MPV (<11, 11–15, >15 fl), platelet count (<250, 250–400, >400 × 10^9^/L), and haematocrit (<20, 20–30, >30%). Platelet count and serum iron were also applied as binary and tertiary variables, respectively, based on predefined laboratory reference ranges: thrombocytosis (>484 × 10^9^/L) vs. no thrombocytosis (<484 × 10^9^/L) and normal serum iron (>19 μmol/L) versus functional iron deficiency (serum iron < 19 μmol/L with low transferrin saturation and high TIBC) and absolute iron deficiency (serum iron <19 μmol/L and low or normal TIBC). Reference ranges for transferrin saturation and TIBC were based on predefined external laboratory reference ranges: transferrin saturation: low (<25%), normal (25–52%), high (>52%); TIBC: low (<30 μmol/L), normal (30–70 μmol/L), high (>70 μmol/L). Categorical variables were compared using the Pearson chi^2^ test with post hoc z-testing for significant associations. Following visual assessment for normality using Q-Q plots, continuous data was assessed using Mann–Whitney, Kruskal–Wallis, and the Spearman correlation. Quade non-parametric analysis of covariance was applied for multivariable analysis, and Bonferroni adjustment of significance was used to account for pairwise comparisons. The results are expressed as median, interquartile range (IQR), and min–max, and significance was set at *p* < 0.05.

## 3. Results

### 3.1. Descriptive Statistics

A total of 151 dogs with iron profile and concurrent CBC results were identified. Four dogs were excluded due to a diagnosis of immune-mediated thrombocytopenia. One was excluded due to pancytopenia with severe thrombocytopenia, and another due to sampling post-blood transfusion. Four dogs (all Cavalier King Charles Spaniels) were excluded due to high breed incidence of congenital macrothrombocytopaenia. Consequently, 141 dogs were included in the final analysis, of which 57 (40.4%) were female (9 entire, 48 neutered) and 83 (58.8%) were male (10 entire and 73 neutered). The sex of one dog was not recorded. The median age was 8 years (min 0.3, max 15). The study population included 51 breeds, only 4 of which comprised >5 dogs: Labrador Retriever (n = 21), Cocker Spaniel (n = 12), Staffordshire Bull Terrier (n = 7), and Border Terrier (n = 6). Of the 141 dogs, 120 (85.1%) dogs were anaemic and 27 (19.1%) had thrombocytosis. In total, 86 (61%) dogs had low serum iron concentration; 56/86 (67%) with functional and 30/86 (36%) with absolute iron deficiency. Of the 86 dogs with iron deficiency, 77 (90%) were anaemic (functional n = 50, absolute n = 27) and 19 (22%) had thrombocytosis (functional n = 10, absolute n = 9). The large number of included breeds precluded statistical analysis, but of the breeds that were over-represented, 11/21 (52.4%) Labrador Retrievers, 10/12 (83.3%) Cocker Spaniels, 7/7 (100%) Bull Terriers, and 4/6 (66.7%) Border Terriers had low serum iron concentrations. The total number of dogs and proportions with iron deficiency and thrombocytosis according to signalment, affected body system, disease pathology, and blood results are summarised in Table 1. Of the 71 dogs with GI disease, 30 (42%) were diagnosed with chronic inflammatory enteropathy and 22 (31%) with GI neoplasia. Other diagnoses included drug-related GI bleeding (n = 4), exocrine pancreatic insufficiency (n = 3), GI foreign bodies (n = 3), idiopathic gastritis (n = 3), parasitism (n = 1), malnutrition (n = 1), and GI bleeding of unknown aetiology (n = 4). Other disease categories included hepatic (congenital hepatic abnormalities n = 6, hepatic neoplasia n = 3, hepatitis n = 1), renal (renal neoplasia n = 6, chronic kidney disease n = 3, idiopathic renal haematuria n = 2), haematologic (immune-mediated haemolytic anaemia n = 11, precursor-targeted immune-mediated anaemia n = 12, haematopoeitic malignancy n = 5, myelofibrosis n = 3), and other (CNS disease n = 7, endocrinopathies n = 6, pancreatitis n = 1).

### 3.2. Platelet Parameter and Serum Iron Concentration Analysis

The distribution of continuous platelet count data differed significantly between dogs with and without iron deficiency (MW-U 3007.5, SE 236.538, standard t 2.716, *p* = 0.007); however, the distribution of continuous serum iron data did not differ between dogs with and without thrombocytosis (MW-U 1252.5, SE 190.764, standard t −1.507, *p* = 0.132). In univariable analysis, platelet count categories differed across iron deficiency categories (*p* = 0.036), with higher platelet count categories being more common in dogs with both functional and absolute iron deficiency than in dogs with normal serum iron concentrations (Table 1). The prevalence of thrombocytosis also differed across serum iron categories (*p* = 0.017) (Table 1), being more common in dogs with serum iron ≤ 20 μmol/L than in those with serum iron > 20 μmol/L (Table 1). In multivariable analysis, platelet count category was associated with serum iron concentration category (*p* = 0.013), but not with the iron deficiency category (*p* = 0.179) independent of sex, age, haematocrit, body system, and pathology type; dogs with serum iron concentrations < 10 μmol/L differed from those with serum iron of 10–20 μmol/L (*p* = 0.014) and >20 μmol/L (*p* = 0.008) (Table 2). No associations were found for age, sex, serum cobalamin, or haematocrit categories with iron deficiency (Table 1).

**Figure 1:**
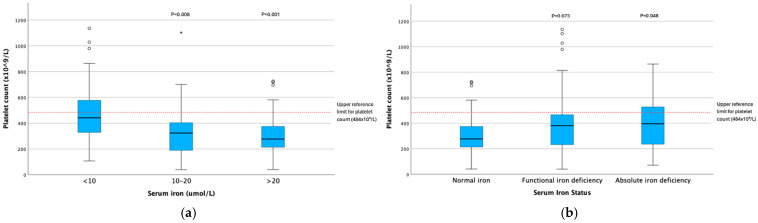
Box and whisker plots illustrating platelet count distributions across serum iron concentration (**a**) and iron deficiency (**b**) categories (*p*-values represent significance in comparison with <10 μmol/L iron concentration and normal serum iron categories, respectively). Circles represent mild outliers (<1.5× IQR) and asterisks represent extreme outliers (>1.5× IOR).

When analysed as a continuous variable, platelet count distribution was different across iron concentration categories (*p* < 0.001), with dogs with serum iron < 10 μmol/L having higher platelet counts than those in the 10–20 μmol/L (*p* = 0.008) and >20 μmol/L (*p* = 0.001) categories (Table 3) (Figure 1a). This relationship was maintained independent of sex, age, haematocrit, body system, and pathology type (*p* = 0.003), with dogs with serum iron < 10 μmol/L having higher platelet counts than those with iron concentrations of 10–20 μmol/L (*p* = 0.002) and >20 μmol/L (*p* = 0.005) (Table 2). Platelet count distribution was also different across iron deficiency categories (*p* = 0.022); dogs with absolute (*p* = 0.048), but not with functional (*p* = 0.073) iron deficiency, had higher platelet counts than those with normal serum iron (Table 4) (Figure 1b). However, this relationship was not maintained independent of sex, age, haematocrit, body system, and pathology type (*p* = 0.263) (Table 5). The distribution of haematocrit differed across serum iron concentration categories (*p* = 0.021), but not across iron deficiency categories (*p* = 0.873); dogs with serum iron < 10 μmol/L had lower haematocrits than those with serum iron > 20 μmol/L (*p* = 0.047) (Table 3 and Table 4). There was no difference in platelet count between dogs with mild (Hct 27–37%, n = 43), moderate (Hct 17–27%, n = 40), and severe (Hct < 17%, n = 39) anaemia in the current study in either univariable (t = 3.191, df 3, *p* = 3.191) or in multivariable analyses independent of sex, age, serum iron, body system, and pathology type (F 0.667, dfh 3, dfe 131, *p* = 0.574). No association was found between MPV or serum cobalamin with iron categories (Table 3 and Table 4). A weak but significant negative correlation was found between serum iron concentration and platelet count (Spearman’s rho −0.244, CI −0.398 to −0.77, *p* = 0.004).

### 3.3. Disease Pathology and Serum Iron Concentration Analysis

In the univariable analysis, iron deficiency (*p* < 0.001) and thrombocytosis (*p* < 0.001) were significantly associated with pathology type and affected body system (Table 1). Gastrointestinal disease was more prevalent in dogs with both functional and absolute iron deficiency than in those without, and dogs with functional iron deficiency were more likely to have neoplasia than those without. In multivariable analysis, neoplasia remained significant across serum iron concentration categories (*p* < 0.001), but not across iron deficiency categories (*p* = 0.434) independent of sex, age, haematocrit, platelet count, and body system, with neoplasia being more common in dogs with serum iron < 10 μmol/L than in those with serum iron 10–20 μmol/L (*p* < 0.001) and >20 μmol (*p* = 0.001) (Table 5). When assessed as binary variables, neoplasia was more common in dogs with iron deficiency than those without (*p* < 0.001, chi^2^ 15.070, df 1), with an odds ratio of 5.1 (CI 2.1–12.2, *p* < 0.001). The associations of platelet count with pathology type were not sustained in the multivariable analysis independent of sex, age, haematocrit, serum iron, and body system (Table 5).

**Figure 2:**
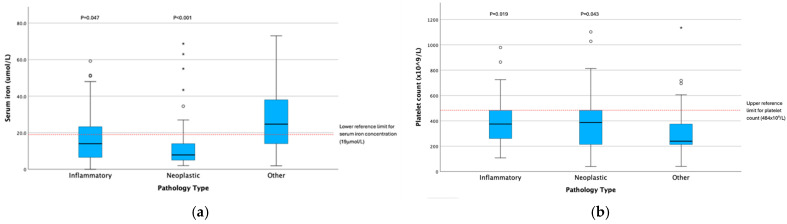
Box and whisker plots illustrating serum iron concentration (**a**) and platelet count (**b**) distributions across disease pathology categories (*p*-values represent significance in comparison with ‘other’ pathology type category). Circles represent mild outliers (<1.5× IQR) and asterisks represent extreme outliers (>1.5× IOR).

When assessed as continuous variables, serum iron concentration was significantly lower (*p* < 0.001) and platelet count was significantly higher (*p* = 0.012) in dogs with GI disease compared with diseases affecting other body systems (Table 6). However, these relationships were not sustained in multivariable analysis independent of sex, age, haematocrit, and pathology type (Table 5). Serum iron concentrations were lower (*p* < 0.001) (Figure 2a) and platelet counts were higher (*p* = 0.010) (Figure 2b) in dogs with neoplasia than in dogs with other disease pathologies (Table 7); serum iron concentration (*p* = 0.001), but not platelet count (*p* = 0.698), remained significant independent of sex, age, haematocrit, and body system (Table 5).

## 4. Discussion

As hypothesised, the results suggest that, as in humans, platelet count increases with both functional and absolute iron deficiency in dogs. As in humans, this effect was modest in the majority of dogs, with iron deficiency uncommonly being associated with overt thrombocytosis (platelet count > 484 × 10^9^/L). As a result, the prevalence of overt thrombocytosis did not differ between dogs with and without iron deficiency; however, thrombocytosis was more common in dogs with serum iron < 10 μmol/L, suggesting that the degree of platelet elevation correlates with the magnitude rather than the type of iron deficiency. Indeed, although weak (Spearman’s rho −0.244), a significant negative correlation was confirmed between serum iron concentration and platelet count (*p* = 0.004). The prevalence of thrombocytosis of 22.1% (17.9% in functional and 30.0% in absolute iron deficiency) found in this study is comparable to the 10–30% prevalence of thrombocytosis reported in iron deficient humans [10,13,20]. Due to the diverse aims, study designs, and result reporting, it is not possible to derive comparable prevalence data from previously published canine studies; however, thrombocytosis has been described in dogs with low serum iron concentration [5] and has been documented in 31.8% of dogs with chronic enteritis, 18% of whom were anaemic [2].

In humans, marked platelet elevations (>950 × 10^9^/L) are reported in 7–13% of patients, and it is this population that have a particularly high risk of thromboembolic complications [18,19,21]. In the current study, platelet counts were extremely variable in the iron-deficient dogs (median 381 × 10^9^/L, IQR 248 × 10^9^/L, min–max 40–1135 × 10^9^/L), but of the dogs with platelet counts above the upper limit of the reference range, 4/27 (11.1%) had severe thrombocytosis (>950 × 10^9^/L). While this suggests an associated thromboembolic risk, the retrospective nature of the study with inherent loss to follow-up precluded any assessment of thromboembolic complications. Thromboembolism has previously been documented in 7.9% of dogs with thrombocytosis [11]; however, prospective studies are required to investigate whether, as in humans, iron-deficiency-related thrombocytosis could have clinically significant consequences.

Although mechanisms for thrombocytosis in iron deficiency remain incompletely understood, they involve altered megakaryopoiesis and platelet phenotype [19,26]. When iron concentration is low, a compensatory increase in the production of progenitor cells favouring the production of platelets over red blood cells predisposes to both anaemia and thrombocytosis. One theory suggests that this might be an evolutionary adaptation to blood loss; the increase in platelet count contributing to haemostasis [27]. In patients with inflammation-associated iron deficiency, the release of cytokines such as interleukin-6 and thrombopoietin further stimulate platelet production [9,28,29]. These mechanisms have not been specifically investigated in dogs; however, in one canine study, thrombocytosis was reported in dogs with mild anaemia, but not in those with moderate-to-severe anaemia [4]. The authors hypothesised that the increased production of platelets in anaemic patients was related to increased erythropoietin production via cross-reaction with thrombopoietin receptors, and that upregulation of erythropoietin receptors in more severe anaemia would negate this effect. Of particular significance, dogs with iron deficiency had higher erythropoietin and lower thrombopoietin concentrations than dogs with normal serum iron. No difference in platelet count was found between dogs with mild, moderate, and severe anaemia in the current study, and future research focusing on the mechanism of thrombocytosis is needed if the pathophysiology is to be elucidated.

In the current study, the vast majority (91.7%) of dogs with iron deficiency were anaemic (90.9% of dogs with functional and 93.1% of dogs with absolute deficiency). This figure is much higher than that reported by Matur et al., who found anaemia in only 77% (24/31) of iron-deficient dogs [5]. Iron deficiency in the absence of anaemia is also reported to be common in humans [13,30,31]. The reason for this discrepancy is uncertain, but a tendency for submission of iron panel tests at a later diagnostic stage in dogs than in humans is a possible explanation. Similarly, the inclusion criteria set out by Matur et al. [5] may have led to inclusion of a higher proportion of dogs with mild or less chronic blood loss. Of the 7/86 dogs with iron deficiency and a normal haematocrit in the current study, GI bleeding was suspected in four, despite an absence of documented melena (ulcerated gastric carcinoma n = 2, ileal lymphoma n = 1, portosystemic shunting and pancreatitis n = 1). The other three dogs had documented haematuria (renal transitional cell carcinoma n = 1, multiple myeloma and urinary tract infection n = 1, idiopathic renal haematuria n = 1).

In the current study, where submission of iron panel tests was decided based on clinician perception of clinical need, 71/141 (50.4%) were requested in dogs with GI disease. Due to its large surface area and propensity to ulceration, the GI tract is a common site for chronic blood loss; it is therefore not surprising that iron deficiency is well described in dogs with chronic enteropathy [2,6]. However, significant GI blood loss may occur with only mild clinical signs and may go unnoticed for extended periods in the absence of obvious melena or associated haematologic abnormalities [6,32]. Indeed, in humans, occult inflammatory enteropathies are a frequent finding in patients with iron deficiency anaemia of unknown origin [33]. Undiagnosed chronic enteropathy is therefore the most likely explanation for dogs with unexplained iron-deficiency anaemia. In this study, twenty-nine dogs without diagnosed GI disease had iron deficiency, only nine of which had absolute deficiency; three had documented urinary tract haemorrhage but it is conceivable that the remaining six had occult GI haemorrhage. Given this propensity for blood loss, it was not surprising that, in univariable analysis, serum iron concentrations were lower in dogs with GI disease compared with other affected body systems. Interestingly, this relationship was not maintained independent of age, sex, haematocrit, and pathology type.

Iron deficiency is common in the setting of human neoplasia, occurring in 29–42% of patients with malignancies, particularly solid tumours and haematologic malignancies [30,33]. Of the dogs with neoplasia in the current study, 35/42 (83.3%) had iron deficiency and 35/86 (40.7%) dogs with iron deficiency had neoplasia. Moreover, independent of age, sex, haematocrit, and affected body system, serum iron concentration was lower in dogs with neoplasia, and dogs with neoplasia were more likely to have serum iron concentrations < 10 μmol/L. In human malignancies, the lowering of serum iron is predominantly attributed to functional rather than absolute deficiency [34]. Mirroring this, 28/42 (66.7%) dogs with neoplasia in the current study had functional iron deficiency, while only 7/42 (16.7%) had absolute iron deficiency. Nevertheless, while an over-representation of functional but not absolute iron deficiency in dogs with neoplasia was confirmed in univariable analysis, this relationship was not borne out independent of age, sex, haematocrit, platelet count, and body system.

Previous studies have also documented an association between neoplasia and thrombocytosis in dogs, especially with marked platelet count elevations [11,22,23]. While they did not consider iron deficiency, Athanasiou et al. reported that mean platelet counts in anaemic dogs with neoplasia were significantly higher than in dogs with other disease aetiologies [12]. In the current study, the prevalence of thrombocytosis did not differ between disease pathology types, and although univariable analysis identified higher platelet counts in dogs with both neoplastic and inflammatory disease compared with other disease pathologies, this relationship was not sustained independent of age, sex, haematocrit, serum iron and body system. More studies are necessary to determine the prevalence, clinical significance, prognostic and therapeutic utility of thrombocytosis in dogs with cancer, but it is clear that in dogs with anaemia and thrombocytosis, neoplasia should be included in the list of differential diagnoses. It is also of note that thrombocytosis is associated with the use of corticosteroids and antineoplastic agents [11,22]; thus, the lack of consideration of medications received by dogs in the current study could have impacted the results. Similarly, due to the retrospective nature of the study, the implications of concurrent disease for both the platelet count and iron panel results may not have been comprehensively documented.

Another limitation is that, despite a reasonable sample size, some subgroups remained small, in some cases, precluding assessment, and in others conferring an increased risk of type II error. For example, in addition to iron, cobalamin deficiency also influences platelet and red blood cell production [35] and is a common feature of gastrointestinal disease in dogs [36]. As such, cobalamin represents an important confounder in this study. While included in the multivariable analysis, serum cobalamin concentration data was available for only 48/141 dogs; thus, the negation of its potential effects cannot be guaranteed. Similarly, in humans, differences in platelet count are well documented based on age, sex, and reproductive status [37,38,39]. While no associations with age or sex were found in the study cohort, the number of entire dogs was too low to assess for any effect of neuter status. Finally, the retrospective methodology and long study period also pose potential limitations. Although the same laboratories and test methodologies were used for iron and haematology parameters, respectively, throughout the study, analyser upgrades and/or reference range changes may have been implemented during this time, and sample storage conditions and storage times are likely to have varied. The effects of both are expected to be minimal but, nevertheless, introduce the possibility of suboptimal data comparability. Coherent assessment of follow-up data was also not possible, and future studies should address changes in iron and platelet parameters in response to treatment of the underlying disease.

## 5. Conclusions

A weak but significant negative correlation was confirmed between serum iron concentration and platelet count, and a significant negative association between serum iron concentration and platelet count was maintained independent of age, sex, haematocrit, affected body system, and pathology type. Platelet counts did not differ between dogs with functional versus absolute iron deficiency, but, independent of age, sex, haematocrit, platelet count, and body system, serum iron concentrations were lower in dogs with neoplasia than in those with other disease pathologies. In conclusion, thrombocytosis appears to be associated with both functional and absolute iron deficiency in dogs, and preferential reduction in serum iron concentration in dogs with neoplasia highlights the need to consider iron panels in these patients. Additional studies are required to elucidate the clinical relevance of iron-deficiency-related thrombocytosis in dogs, in particular, whether it carries diagnostic, monitoring, prognostic, or therapeutic utility.

## Figures and Tables

**Table 1:** Comparisons of serum iron status and thrombocytosis across signalment, disease, haematology and serum biochemistry subcategories.

Variable	Category	Total No. (%) of Dogs	No. (%) Dogs in Serum Iron Categories			Pearson Chi^2^ Comparisons for Iron Deficiency Categories		No. (%) of Dogs with Thrombocytosis (Plt > 484 × 10^9^/L)	Pearson Chi^2^ Comparisons for Thrombocytosis Categories	
			Normal Serum Iron	Functional Iron Deficiency	Absolute Iron Deficiency	t (df)	*p*		t (df)	*p*
Sex	Female	57 (40.7)	23 (42.6)	23 (41.1)	11 (36.7)	0.286 (2)	0.867	10 (37.0)	0.187 (1)	0.665
	Male	83 (59.3)	31 (57.4)	33 (58.8)	19 (63.3)			17 (63.0)		
	Unknown	1	-	-	-			1		
Age (years)	<6	40 (28.6)	14 (25.9)	19 (33.9)		3.271 (4)	0.514	9 (33.3)	2.195 (2)	0.334
	6–10	59 (42.1)	26 (48.1)	22 (39.3)				8 (29.6)		
	>10	41 (29.3)	14 (25.9)	15 (26.8)				10 (37.0)		
	Unknown	1	-	-	-			1		
Affected Body System	Gastrointestinal	71 (51.8)	16 (30.2)	35 (63.6)	20 (69.0)	16.423 (2)	**<0.001** *^1^	18 (66.7)	2.967 (1)	0.085
	Other	66 (48.2)	37 (69.8)	20 (36.4)	9 (31.0)			9 (33.3)		
	Unknown	4	-	-	-			4		
Pathology Type	Inflammatory	45 (32.8)	14 (26.4)	16 (29.1)	15 (51.7)	30.671 (4)	**<0.001** *^2^	10 (37.0)	1.656 (2)	0.437
	Neoplastic	42 (30.7)	7 (13.2)	28 (50.9)	7 (24.1)			10 (37.0)		
	Other	50 (36.5)	32 (60.4)	11 (20.0)	7 (24.1)			7 (25.9)		
	Unknown	4	-	-	-			4		
Serum B12 (ng/L)	<300	22 (47.8)	7 (38.9)	12 (57.1)	3 (2.9)	1.376 (2)	0.503	3 (50.0)	0.013 (1)	0.909
	>300	24 (52.2)	11 (61.1)	9 (42.9)	4 (57.1)			3 (50.0)		
	Unknown	95	-	-	-			95		
Haematocrit (%)	<20	54 (38.8)	24 (43.6)	20 (36.4)	10 (34.5)	8.218 (4)	0.084	11 (42.3)	3.404 (2)	0.182
	20–30	44 (31.7)	11 (20.0)	24 (43.6)	9 (31.0)			11 (42.3)		
	>30	41 (29.5)	20 (36.4)	11 (20.0)	10 (34.5)			4 (5.4)		
	Unknown	2	-	-	-			2		
Platelet Count (×10^9^/L)	<250	50 (35.5)	26 (47.3)	16 (28.6)	8 (26.7)	10.248 (4)	**0.036** *^3^	-	-	-
	250–400	42 (29.8)	18 (32.7)	17 (30.4)	7 (23.3)			-		
	400	49 (34.8)	11 (20.0)	23 (41.1)	15 (50.0)			-		
	Unknown	0	-	-	-			-		
MPV (fl)	<11	29 (32.2)	11 (31.4)	13 (35.1)	5 (27.8)	0.411 (4)	0.982	10 (52.6)	-	-
	11–15	32 (35.6)	13 (37.1)	12 (32.4)	7 (38.9)			5 (26.3)		
	>15	29 (32.2)	11 (31.4)	12 (32.4)	6 (33.3)			4 (21.1)		
	Unknown	51	-	-	-			51		
TIBC (μmol/L)	<55	50 (36.8)	18 (36.0)	32 (57.1)	0	-	-	6 (22.2)	3.138 (2)	0.208
	55–70	43 (31.6)	19 (38.0)	24 (42.9)	0			10 (37.0)		
	>70	43 (31.6)	13 (26.0)	0	30 (100)			11 (40.7)		
	Unknown	5	-	-	-			5		
Transferrin Saturation (%)	<25	64 (47.4)	0	37 (66.1)	27 (90.0)	-	-	16 (59.3)	1.901 (1)	0.168
	>25	71 (52.6)	49 (100)	19 (3.9)	3 (10.0)			11 (40.7)		
	Unknown	6	-	-	-			6		
Serum iron concentration (μmol/L)	<10	48 (34.0)	0	31 (55.4)	17 (56.7)	-	-	15 (55.6)	8.207 (2)	**0.017** *^4^
	10–20	40 (28.4)	5 (9.1)	25 (44.6)	13 (43.3)			3 (11.1)		
	>20	53 (37.6)	50 (90.9)	0	-			9 (33.3)		
	Unknown	0	-	-	-			0		

MPV = mean platelet volume, TIBC = total iron binding capacity, Plt = platelet count, t = test statistic, df = degrees of freedom, *p* = significance at <0.05. *^1^ Functional and absolute iron deficiency groups: gastrointestinal disease overrepresented, Normal serum iron group: gastrointestinal disease underrepresented. *^2^ Normal serum iron and absolute iron deficiency groups: even distribution of inflammatory, neoplastic and other pathology types, Functional iron deficiency: over representation of neoplastic subgroup. *^3^ Normal serum iron group: even distribution of Plt subgroups, Functional and absolute iron deficiency: overrepresentation of Plt > 400 subgroup. *^4^ Normal/low Plt group: even distribution of iron concentration subgroups, Thrombocytosis group: underrepresentation of serum iron > 20 subgroup.

**Table 2:** Multivariable analysis between serum iron and platelet data (a).

Variables	Subcategories	t	DFH/DFE	*p*	Post-hoc Comparisons	t (df)	*p*
Serum iron concentration category (μmol/L) vs. platelet count category	<10	4.526	2/132	**0.013** *^1^	<10 vs. 10–20	2.489 (132)	**0.014**
	10–20				<10 vs. >20	2.678 (132)	**0.008**
	>20				10–20 vs. >20	0.005 (132)	0.996
Iron deficiency category vs. platelet count category	None	1.741	2/132	0.179 *^1^	-	-	-
	Functional				-	-	-
	Absolute				-	-	-
Serum iron concentration category (μmol/L) vs. continuous platelet count	<10	6.185	2/132	**0.003** *^1^	<10 vs. 10–20	3.173 (132)	**0.002**
	10–20				<10 vs. >20	2.854 (132)	**0.005**
	>20				10–20 vs. >20	−0.522 (132)	0.603
Iron deficiency category vs. continuous platelet count data	None	1.349	2/132	0.263 *^1^	-	-	-
	Functional				-	-	-
	Absolute				-	-	-

Plt = platelet count, t = test statistic, DFH = hypothesis degrees of freedom, DFE = effective degrees of freedom, *p* = significance at <0.05, df = degrees of freedom. *^1^ Independent of sex, age, haematocrit, body system and pathology type.

**Table 3:** Univariable comparison of continuous platelet count, MPV, haematocrit and serum B12 concentration data across serum iron deficiency categories.

Variable	Raw Data				KW Comparisons			Post-hoc Comparisons					
	Serum Iron Category (μmol/L)	Number of Dogs	Median	IQR (Min–Max)	Number of Dogs in Analysis	t (df)	*p*	Category Comparisons Between Iron Subgroups	t	SE	Standard t	*p*	Adj. *p*
Haematocrit (l/l)	<10	47	20.7	12 (8–40)	139	7.714 (2)	**0.021**	<10 vs. 10–20	–20.717	8.722	−2.375	**0.018**	0.053
	10–20	39	28.3	17 (10–44)				<10 vs. >20	–19.519	8.068	−2.419	**0.016**	**0.047**
	>20	53	26.3	20 (7–80)				10–20 vs. >20	1.197	8.495	0.141	0.888	1.000
Platelet Count × 10^9^/L	<10	48	442	266 (108–1135)	141	14.726 (2)	**<0.001**	<10 vs. 10–20	26.423	8.743	3.022	**0.003**	**0.008**
	10–20	40	324	225 (40–1102)				<10 vs. >20	28.804	8.137	3.540	**<0.001**	**0.001**
	>20	53	277	162 (41–725)				10–20 vs. >20	2.381	8.554	0.278	0.781	1.000
MPV (fl)	<10	30	14.5	6.8 (7.9–20.1)	90	2.172 (2)	0.338	-	-	-	-	-	-
	10–20	27	11.5	5.6 (6.3–18.7)				-	-	-	-	-	-
	>20	33	12.4	6.5 (8.1–23.8)				-	-	-	-	-	-
Serum B12 (pg/mL)	<10	14	245	137 (125–965)	46	5.755 (2)	0.056	-	-	-	-	-	-
	10–20	16	310	248 (74–1359)				-	-	-	-	-	-
	>20	16	373	376 (142–1523)				-	-	-	-	-	-

MPV = mean platelet volume, IQR = interquartile range, KW = Kruskal Wallis, t = test statistic, df = degrees of freedom, SE = standard error, *p* = significance at <0.05, Adj. *p* = adjusted significance.

**Table 4:** Univariable comparison of continuous platelet count, MPV, haematocrit and serum B12 concentration data across serum iron concentration categories.

Variable	Raw Data				KW Comparisons			Post-hoc Comparisons					
	Iron Deficiency Category	Number of Dogs	Median	IQR (Min–Max)	Number of Dogs in Analysis	t (df)	*p*	Category Comparisons Between Iron Subgroups	t	SE	Sdandard t	*p*	Adj. Sig.
Haematocrit (l/l)	None	55	24.9	20 (7–80)	139	0.355 (2)	0.837	-	-	-	-	-	-
	Functional	55	24.0	11 (10–44)				-	-	-	-	-	-
	Absolute	29	25.6	19 (8–41)				-	-	-	-	-	-
Platelet Count × 10^9^/L	None	55	277	162 (41–725)	141	7.654 (2)	**0.022**	None vs. Functional	–17.458	7.753	2.252	**0.024**	0.073
	Functional	56	381	239 (40–1135)				None vs. Absolute	–22.315	9.269	2.407	**0.016**	**0.048**
	Absolute	30	396	298 (71–864)				Functional vs. Absolute	–4.857	9.240	0.526	0.599	1.000
MPV (fl)	None	35	12.4	7 (6–24)	90	0.395 (2)	0.873	-	-	-	-	-	-
	Functional	37	12.8	7 (8–20)				-	-	-	-	-	-
	Absolute	18	13.5	5 (8–19)				-	-	-	-	-	-
Serum B12 (pg/mL)	None	18	332	331 (100–1523)	46	2.419 (2)	0.298	-	-	-	-	-	-
	Functional	21	277	195 (74–1359)				-	-	-	-	-	-
	Absolute	7	506	755 (125–1322)				-	-	-	-	-	-

MPV = mean platelet volume, IQR = interquartile range, KW = Kruskal Wallis, t = test statistic, df = degrees of freedom, SE = standard error, *p* = significance at <0.05, Adj. *p* = adjusted significance.

**Table 5:** Multivariable analysis between body system and pathology categories.

Variables	Subcategories	t	DFH/DFE	*p*	Post-hoc Comparisons	t (df)	*p*
Body system vs. serum iron concentration category	Gastrointestinal	2.147	1/133	0.145 *^1^	-	-	-
	Other				-	-	-
Body system vs. iron deficiency category	Gastrointestinal	1.302	1/133	0.256 *^1^	-	-	-
	Other				-	-	-
Body system vs. continuous serum iron data	Gastrointestinal	3.626	1/133	0.059 *^1^	-	-	-
	Other				-	-	-
Body system vs. platelet category	Gastrointestinal	0.006	1/133	0.939 *^2^	-	-	-
	Other				-	-	-
Body system vs. continuous platelet count	Gastrointestinal	0.000	1/133	0.985 *^2^	-	-	-
	Other				-	-	-
Pathology type vs. serum iron concentration category	Inflammatory	8.449	2/132	**<0.001** *^3^	Serum iron <10 vs. 10–20	3.733 (132)	**<0.001**
	Neoplastic				Serum iron 10–20 vs. >20	0.448 (132)	0.655
	Other				Serum iron <10 vs. >20	−3.423 (132)	**0.001**
Pathology type vs. iron deficiency category	Inflammatory	0.841	2/132	0.434 *^3^	-	-	-
	Neoplastic				-	-	-
	Other				-	-	-
Pathology type vs. continuous serum iron data	Inflammatory	7.449	2/132	**0.001** *^3^	Inflammatory vs. neoplastic	3.657 (132)	**<0.001**
	Neoplastic				Inflammatory vs. other	0.827 (132)	0.410
	Other				Neoplastic vs. other	−2.969 (132)	**0.004**
Pathology type vs. platelet count category	Inflammatory	0.668	2/132	0.515 *^4^	-	-	-
	Neoplastic				-	-	-
	Other				-	-	-
Pathology type vs. continuous platelet count data	Inflammatory	0.360	2/132	0.698 *^4^	-	-	-
	Neoplastic				-	-	-
	Other				-	-	-

Plt = platelet count, t = test statistic, DFH = hypothesis degrees of freedom, DFE = effective degrees of freedom, *p* = significance at <0.05, df = degrees of freedom. *^1^ Independent of sex, age, haematocrit, platelet count and pathology type. *^2^ Independent of sex, age, haematocrit, serum iron and pathology type. *^3^ Independent of sex, age, haematocrit, platelet count and body system. *^4^ Independent of sex, age, haematocrit, serum iron and body system.

**Table 6:** Univariable comparison of continuous platelet count and serum iron concentration data across body system categories.

Variable	Raw Data				MW Comparisons				
	Body System Category	n	Median	IQR (Min–Max)	Number of Dogs in Analysis	t	SE	Standard t	*p*
Serum iron concentration (μmol/L)	Gastrointestinal	71	11	14 (0–59)	137	3234.5	232.029	3.842	**<0.001**
	Other	66	22	29 (2–73)					
Platelet Count × 10^9^/L	Gastrointestinal	71	375	288 (40–1102)	137	1763	232.092	−2.499	**0.012**
	Other	66	315	191 (41–1135)					

n = number of dogs, IQR = interquartile range, MW = Mann Whitney, t = test statistic, SE = standard error, *p* = significance at *p* < 0.05.

**Table 7:** Univariable comparison of continuous platelet count and serum iron concentration data across disease pathology categories.

Variable	Raw Data				KW Comparisons			Post-hoc Comparisons					
	Disease Pathology Category	n	Median	IQR (Min-Max)	Number of Dogs in Analysis	t (df)	*p*	Category Comparisons Between Iron Subgroups	t	SE	Standard t	*p*	Adj. *p*
Serum iron concentration (μmol/L)	Inflammatory	45	14	17.4 (0–59)	137	19.178 (2)	**<0.001**	N-O	−36.177	8.304	−4.357	<0.001	**<0.001**
	Neoplastic	42	8	9 (2–69)				I-O	−19.726	8.152	−2.420	0.016	**0.047**
	Other	50	25	24 (2–73)				N-I	16.452	8.512	1.933	0.053	0.160
Platelet Count × 10^9^/L	Inflammatory	45	375	227 (108–979)	137	9.232 (2)	**0.010**	N-O	20.324	8.306	2.447	0.014	**0.043**
	Neoplastic	42	387	283 (40–1102)				I-O	22.283	8.154	2.733	0.006	**0.019**
	Other	50	240	164 (41–1135)				N-I	1.960	8.514	0.230	0.818	1.000

n = number of dogs, IQR = interquartile range, KW = Kruskal Wallis, t = test statistic, SE = standard error, *p* = significance at *p* < 0.05, Adj. *p* = adjusted significance.

## Data Availability

The original contributions presented in this study are included in the article. Further inquiries can be directed to the corresponding author.

## Abbreviations

The following abbreviations are used in this manuscript:

CBC	Complete blood count
MPV	Mean platelet volume
TIBC	Total iron binding capacity
ME	Male entire
MN	Male neutered
FE	Female entire
FN	Female neutered
GI	Gastrointestinal
IQR	Interquartile range
CI	Confidence interval
SE	Standard error
t/F/H/U	Test statistics (Pearson Chi^2^/Quade-ANOVA/Kruskal–Wallis/Mann–Whitney)
df	Degrees of freedom
DFH	Hypothesis degrees of freedom
DFE	Effective degrees of freedom
MW	Mann–Whitney
KW	Kruskal–Wallis

## References

[1] Moreno C.J.A., Romero C.M.S., Gutiérrez M.M. (2009). Classification of anemia for gastroenterologists. World J. Gastroenterol..

[2] Marchetti V., Lubas G., Lombardo A., Corazza M., Guidi G., Cardini G. (2010). Evaluation of erythrocytes, platelets, and serum iron profile in dogs with chronic enteropathy. Vet. Med. Int..

[3] McCown J.L., Specht A.J. (2011). Iron homeostasis and disorders in dogs and cats: A review. J. Am. Anim. Hosp. Assoc..

[4] Naigamwalla D.Z., Webb J.A., Giger U. (2012). Iron deficiency anemia. Can. Vet. J..

[5] Matur E., Ekiz E.E., Erek M., Ergen E., Kucuk S.H., Erhan S., Özcan M. (2019). Relationship between anemia, iron deficiency, and platelet production in dogs. Med. Weter..

[6] Ristic J.M., Stidworthy M.F. (2002). Two cases of severe iron-deficiency anaemia due to inflammatory bowel disease in the dog. J. Small Anim. Pract..

[7] Bohn A.A. (2013). Diagnosis of disorders of iron metabolism in dogs and cats. Vet. Clin. N. Am. Small Anim. Pract..

[8] Feldman B.F., Keen C.L., Kaneko J.J., Farver T.B. (1981). Anemia of inflammatory disease in the dog: Measurement of hepatic superoxide dismutase, hepatic nonheme iron, copper, zinc, and ceruloplasmin and serum iron, copper, and zinc. Am. J. Vet. Res..

[9] Feldman B.F., Kaneko J.J., Farver T.B. (1981). Anemia of inflammatory disease in the dog: Availability of storage iron in inflammatory disease. Am. J. Vet. Res..

[10] Rokkam V.R., Killeen R.B., Kotagiri R. (2025). Secondary Thrombocytosis.

[11] Neel J.A., Snyder L., Grindem C.B. (2012). Thrombocytosis: A retrospective study of 165 dogs. Vet. Clin. Pathol..

[12] Athanasiou L.V., Polizopoulou Z.S., Papavasileiou E.G., Mpairamoglou E.L., Kantere M.C., Rousou X.A. (2017). Magnitude of reactive thrombocytosis and associated clinical conditions in dogs. Vet. Rec..

[13] Mhadgut H., Galadima H., Tahhan H.R. (2018). Thrombocytosis in Iron Deficiency Anemia. Blood.

[14] Voudoukis E., Karmiris K., Oustamanolakis P., Theodoropoulou A., Sfiridaki A., Paspatis G.A., Koutroubakis I.E. (2013). Association between thrombocytosis and iron deficiency anemia in inflammatory bowel disease. Eur. J. Gastroenterol. Hepatol..

[15] Kulnigg-Dabsch S., Schmid W., Howaldt S., Stein J., Mickisch O., Waldhor T., Evstatiev R., Kamali H., Volf I., Gasche C. (2013). Iron deficiency generates secondary thrombocytosis and platelet activation in IBD: The randomized, controlled thromboVIT trial. Inflamm. Bowel Dis..

[16] Kawasugi K., Yamamoto T., Shirafuji N., Oka Y. (2014). Increased Levels of Thrombopoietin and IPF in Patients with Iron Deficiency Anemia. Blood.

[17] Keung Y.K., Owen J. (2004). Iron deficiency and thrombosis: Literature review. Clin. Appl. Thromb. Hemost..

[18] Franchini M., Targher G., Montagnana M., Lippi G. (2008). Iron and thrombosis. Ann. Hematol..

[19] Evstatiev R. (2016). Iron deficiency, thrombocytosis and thromboembolism. Wien. Med. Wochenschr..

[20] Song A.B., Kuter D.J., Al-Samkari H. (2020). Characterization of the rate, predictors, and thrombotic complications of thrombocytosis in iron deficiency anemia. Am. J. Hematol..

[21] Yadav K., Yadav V., Margekar S.L., Bansal P., Aggarwal R., Gupta A., Ghotekar L.H. (2023). Iron Deficiency Anemia as a Potential Risk Factor for Unprovoked DVT in Young Patients: A Case Series. J. Assoc. Physicians India.

[22] Hammer A.S. (1991). Thrombocytosis in dogs and cats: A retrospective study. Comp. Haematol. Int..

[23] Woolcock A.D., Keenan A., Cheung C., Christian J.A., Moore G.E. (2017). Thrombocytosis in 715 Dogs (2011–2015). J. Vet. Intern. Med..

[24] Radakovich L.B., Pannone S.C., Truelove M.P., Olver C.S., Santangelo K.S. (2017). Hematology and biochemistry of aging-evidence of “anemia of the elderly” in old dogs. Vet. Clin. Pathol..

[25] Singh M.K., Lamb W.A. (2005). Idiopathic thrombocytopenia in Cavalier King Charles Spaniels. Aust. Vet. J..

[26] Evstatiev R., Bukaty A., Jimenez K., Kulnigg-Dabsch S., Surman L., Schmid W., Eferl R., Lippert K., Scheiber-Mojdehkar B., Kvasnicka H.M. (2014). Iron defiency alters megakaryopoiesis and platelet phenotype independent of thrombopoietin. Am. J. Hematol..

[27] Babikir M., Ahmad R., Soliman A., Al-Tikrity M., Yassin M.A. (2020). Iron-induced thrombocytopenia: A mini-review of the literature and suggested mechanisms. Cureus.

[28] Kadikoylu G., Yavasoglu I., Bolaman Z., Senturk T. (2006). Platelet parameters in women with iron deficiency anemia. J. Natl. Assoc..

[29] Nairz M., Theurl I., Wolf D., Weiss G. (2016). Iron deficiency or anemia of inflammation?: Differential diagnosis and mechanisms of anemia of inflammation. Wien. Med. Wochenschr..

[30] Bouri S., Martin J. (2018). Investigation of iron deficiency anaemia. Clin. Med..

[31] Soppi E.T. (2018). Iron deficiency without anemia—A clinical challenge. Clin. Case Rep..

[32] Pawsat G.A., Fry M.M., Behling-Kelly E., Olin S.J., Schaefer D.M.W. (2023). Bone marrow iron scoring in healthy and clinically ill dogs with and without evidence of iron-restricted erythropoiesis. Vet. Clin. Pathol..

[33] Monzón H., Forné M., González C., Esteve M., Martí J.M., Rosinach M., Mariné M., Loras C., Espinós J.C., Salas A. (2011). Mild enteropathy as a cause of iron-deficiency anaemia of previously unknown origin. Dig. Liver Dis..

[34] Naoum F.A. (2016). Iron deficiency in cancer patients. Rev. Bras. Hematol. Hemoter..

[35] AI Amin A.S.M., Gupta V. (2025). Vitamin B12 (Cobalamin).

[36] Kather S., Grützner N., Kook P.H., Dengler F., Heilmann R.M. (2019). Review of cobalamin status and disorders of cobalamin metabolism in dogs. J. Vet. Intern. Med..

[37] Butkiewicz A., Kemona H., Dymicka-Piekarska V., Matowicka-Karna J., Radziwon P., Lipska A. (2006). Platelet count, mean platelet and thrombocytopoeitic indices in healthy women and men. Thromb. Res..

[38] Bukar A., Waziri G., Buhari M.A., Askira U.M., Stephen L., Obi O.S. (2025). Assessment of platelet counts in women using hormonal contraceptives visiting state specialist hospital Maiduguri, Borno State, Nigeria. Haematol. J. Bangladesh.

[39] Reese J.A., Peck J.D., Deschamps D.R., McIntosh J.J., Knudtson E.J., Terrell D.R., Vesely S.K., George J.N. (2018). Platelet counts during pregnancy. N. Engl. J. Med..

